# Regulatory Effects of an Antioxidant Combination on Seminal Quality and Gut Microbiota in Ningxiang Boars Under Heat Stress

**DOI:** 10.3390/life16010099

**Published:** 2026-01-10

**Authors:** Lu Wang, Cheng Zhang, Siqi Li, Xueer Mei, Xijie Kuang, Qiye Wang, Huansheng Yang

**Affiliations:** Hunan Provincial Key Laboratory of Animal Intestinal Function and Regulation, Hunan International Joint Laboratory of Animal Intestinal Ecology and Health, Laboratory of Animal Nutrition and Human Health, College of Life Sciences, Hunan Normal University, Changsha 410081, China

**Keywords:** antioxidant, Ningxiang boar, sperm motility, semen quality, gut microbiota

## Abstract

Heat stress during summer significantly impairs seminal quality in swine production. As a key genetic resource for enhancing indigenous Chinese fatty pig breeds, Ningxiang boars require effective nutritional strategies to maintain reproductive performance under thermal challenge. This study aimed to investigate the effects of a combined antioxidant dietary supplement on seminal quality, antioxidant status, and gut microbiota in heat-stressed Ningxiang boars. Ten Ningxiang boars were randomly assigned to two groups (n = 5 per group). The control group received a basal diet, while the experimental group was fed the same basal diet supplemented with 400 mg/kg vitamin E, 5 g/kg yeast-derived zinc, 250 mg/kg yeast-derived selenium, and 800 mg/kg N-carbamylglutamate (NCG). Results demonstrated that sperm and seminal plasma superoxide dismutase (SOD) activity was significantly elevated in the supplemented group compared to the control (*p* < 0.05), whereas malondialdehyde (MDA) levels and total antioxidant capacity (T-AOC) did not differ significantly (*p* > 0.05). 16S rRNA gene sequencing revealed that dietary supplementation combined antioxidant markedly altered gut microbiota composition: the abundance of short-chain fatty acid-producing bacteria, particularly members of the *Muribaculaceae* family, increased significantly (*p* < 0.05), while opportunistic pathogens within the *Acholeplasmataceae* family were reduced (*p* < 0.05). These findings suggest that dietary supplementation with this antioxidant combination improves seminal quality in Ningxiang boars, potentially by enhancing endogenous antioxidant defenses and modulating gut microbial balance.

## 1. Introduction

Summer heat stress is a major factor contributing to impaired semen quality in boars, leading to reduced sperm motility and density, as well as increased sperm deformity rates [1]. This seasonal infertility poses a significant economic challenge to pig production. The underlying mechanism is closely linked to oxidative stress; in male animals under thermal challenge, reactive oxygen species (ROS) production exceeds the capacity of endogenous antioxidant defenses, resulting in oxidative damage to spermatozoa membranes and DNA [2,3]. Organisms counteract ROS through antioxidant defense mechanisms [4]. These comprise an enzymatic system, including superoxide dismutase (SOD), glutathione peroxidase (GSH-Px), and catalase (CAT), and a non-enzymatic system involving molecules such as vitamin E, vitamin C, and glutathione [5,6,7,8]. Trace minerals like selenium and zinc are essential cofactors for key antioxidant enzymes (GSH-Px and SOD, respectively) [9,10]. When heat stress overwhelms these endogenous defenses, dietary antioxidant supplementation represents a practical strategy to mitigate oxidative damage and support reproduction [11].

Individual antioxidants have shown promise. Dietary zinc alleviates heat-induced epididymal damage in boars [12], organic selenium enhances GSH-Px activity in semen [4], and vitamin E protects sperm membranes from lipid peroxidation [13]. Furthermore, N-carbamylglutamate (NCG), a synthetic analog of N-acetyl-glutamate, promotes blood ammonia transformation and endogenous arginine synthesis, modulating antioxidant capacity via nitric oxide-dependent pathways [14], with demonstrated benefits for semen quality in poultry [15]. However, the synergistic effects of combining these specific nutrients, particularly in the context of indigenous pig breeds, are less explored.

Emerging evidence suggests a novel link between gut health and systemic physiology, including reproduction. The gut microbiota influences host metabolism, immunity, and inflammation [16]. Notably, diet-induced changes in the microbiota can affect distal organs, but the role of the gut microbiome in mediating the effects of dietary antioxidants on boar reproduction remains largely unknown.

Given that heat stress induces systemic oxidative stress and may disrupt gut homeostasis, we hypothesize that a combined dietary strategy may offer superior benefits. Therefore, this study aimed to evaluate the effects of a dietary combination of vitamin E, yeast-derived zinc, yeast-derived selenium, and NCG on (1) semen quality parameters, (2) seminal antioxidant status (SOD, MDA, T-AOC), and (3) the composition of the gut microbiota in Ningxiang boars under summer heat stress conditions. We posited that the supplement would improve seminal quality not only by directly boosting antioxidant defenses but also potentially through modulating the gut microbial community.

## 2. Materials and Methods

### 2.1. Ethics Statement

The animal care and handling procedures were approved by the Animal Care and Use Committee of Hunan Normal University (Changsha, Hunan, China; Approval No. 2023-011, approved on 27 February 2023). This study utilized only Ningxiang boars to evaluate the potential of an antioxidant combination in improving seminal quality during summer heat stress.

### 2.2. Animals and Experimental Design

Ten healthy, sexually mature Ningxiang boars with confirmed successful breeding histories and no testicular pathology were selected. Obtained in a single batch and matched for age, health status, and body weight, the boars were randomly divided into a control group (Group B, n = 5) and a treatment group (Group A, n = 5). They were individually housed under standardized conditions for a 60-day trial.

Group B received a basal diet formulated to meet the nutritional requirements of mature boars. Group A received the same basal diet supplemented with 400 mg/kg vitamin E, 5 g/kg yeast-derived zinc, 250 mg/kg yeast-derived selenium, and 800 mg/kg NCG. The diet composition and its nutritional levels are presented in Table 1. The trial was conducted at Chuwenxiang Agriculture Co., Ltd. (Changsha, China). All boars received identical routine health management, including a standard vaccination protocol. The study conducted from July to September 2022. The average daily temperatures ranged from 18 °C to 28 °C, with maximum daytime temperatures between 25 °C and 40 °C. Heat stress was functionally defined and confirmed by the observed decline in semen quality parameters (e.g., increased abnormalities) in the control group over the experimental period. Semen was collected before the experiment (T1, baseline), and at 30 (T2) and 60 (T3) days after initiating the experimental diets.

### 2.3. Sample Collection and Semen Quality Assessment

Semen was collected using the false mount technique. Gel-free semen was obtained by filtration through sterile gauze, and volume was recorded immediately. Semen volume, sperm concentration, sperm motility, and sperm morphology were evaluated according to the Chinese National Standard GB 23238-2021 [17]. Ejaculates were centrifuged (3000× *g*, 10 min) to separate seminal plasma and sperm cells; both were stored at −80 °C. Fresh fecal samples were collected via rectal stimulation, placed in sterile tubes on ice, and stored at −80 °C.

### 2.4. Antioxidant Capacity Assays

Malondialdehyde (MDA) levels in sperm and seminal plasma, and seminal plasma total antioxidant capacity (T-AOC), were measured using commercial kits (Nanjing Jiancheng Bioengineering Institute, Nanjing, China). Total superoxide dismutase (SOD) activity in sperm and seminal plasma was determined using kits from Suzhou Kemeing.

### 2.5. 16S rRNA Gene Sequencing and Analysis

Total genomic DNA was extracted from fecal samples using the QIAamp DNA Stool Mini Kit (Qiagen, Hilden, Germany). DNA integrity was verified by 1% agarose gel electrophoresis. V3–V4 region of the bacterial 16S rRNA gene was amplified via PCR using an ABI GeneAmp^®^ 9700 system (Applied Biosystems, Waltham, MA, USA). Amplification products were quantified with a QuantiFluor™-ST fluorometer (Promega, Madison, WI, USA). Sequencing was performed on an Illumina MiSeq platform at Majorbio Bio-Pharm Technology Co., Ltd. (Shanghai, China). Raw sequences were quality-filtered, trimmed, and clustered into operational taxonomic units (OTUs) at 97% similarity. Bioinformatics analysis was conducted using the Majorbio Cloud Platform. Taxonomic assignment was based on the SILVA database (v115). Alpha and beta diversity were assessed using MOTHUR. Treatment effects were tested using PERMANOVA via the Vegan package (v2.5-3). Differential taxa abundance (phylum to genus) was identified using linear discriminant analysis effect size (LEfSe) (LDA score > 3, *p* < 0.05). Robust correlations were defined as |Spearman’s rho| > 0.6 with *p* < 0.01.

### 2.6. Statistical Analysis

Data were analyzed using independent samples *t*-tests in SPSS software 26.0. Results are presented as mean ± standard error of the mean (SEM).

## 3. Results

### 3.1. Semen Quality Parameters

Compared to the control group, the treatment group showed significant increases in sperm density (*p* < 0.05) and sperm motility (*p* < 0.05) at the end of the 60-day experiment period (T3). No significant differences were observed in ejaculate volume or sperm abnormality rate (*p* > 0.05) (Table 2).

### 3.2. Antioxidant Status

SOD activity was significantly higher in both sperm and seminal plasma of Group A compared to Group B (*p* < 0.05). No significant differences were detected in MDA levels or T-AOC (*p* > 0.05) (Table 3).

### 3.3. Gut Microbiota Composition

**Figure 1:**
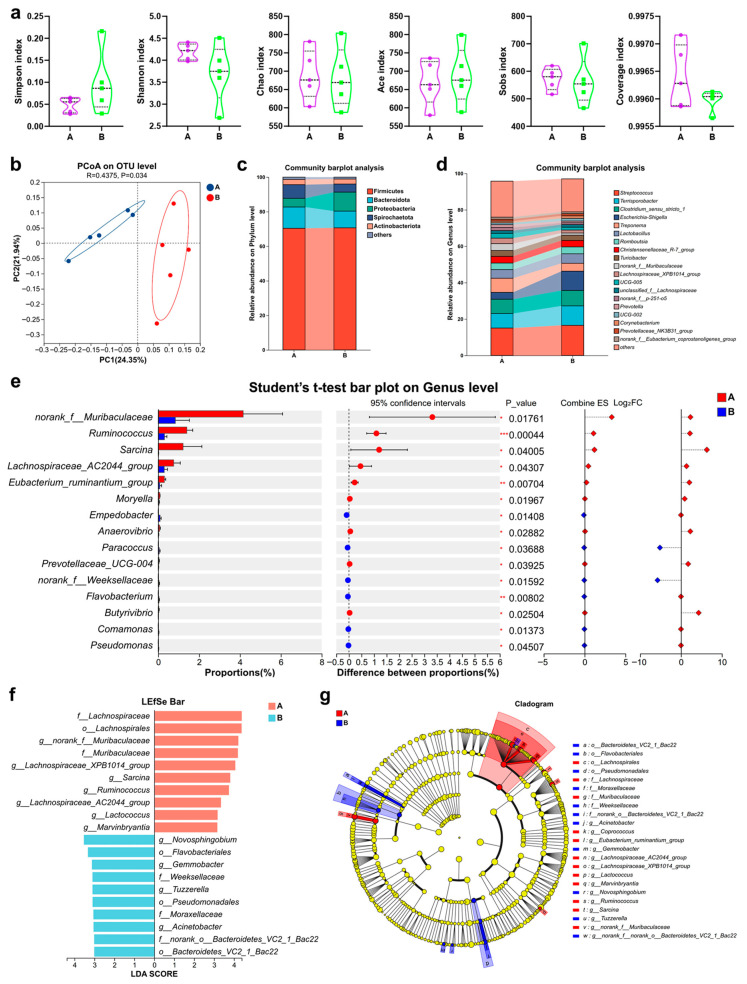
Fecal microbiota composition and taxonomic shifts in Ningxiang boars receiving antioxidant supplementation (n = 5). (**a**). α-diversity. (**b**). β-diversity. (**c**). Microbial composition at the phylum level. (**d**). Microbial composition at the genus level. (**e**). Differentially abundant genera. (**f**,**g**). Linear discriminant analysis effect size (LEfSe) identifying taxa with differential abundance (LDA score ≥ 3). Group A: basal diet supplemented with 400 mg/kg vitamin E, 5 g/kg yeast-derived zinc, 250 mg/kg yeast-derived selenium, and 800 mg/kg NCG; Group B: control (basal diet only). axonomic abbreviations: p_, phylum; c_, class; o_, order; f_, family; g_, genus; s_, species. Data are presented as mean ± SEM. Statistical significance: * *p* < 0.05, ** *p* < 0.01, *** *p* < 0.001 (Student’s *t*-test).

A total of 264,576 high-quality sequences were clustered into 1019 OTUs. Alpha diversity (Ace, Chao1, Shannon) showed no significant differences (*p* > 0.05) (Figure 1a). In contrast, beta diversity analysis (PCoA) revealed a clear separation in microbial community structure between groups (R = 0.4375, *p* = 0.034) (Figure 1b). Dominant phyla included Firmicutes, Bacteroidota, Proteobacteria, Spirochaetota, and Actinobacteriota, with no significant abundance differences (Figure 1c). At the genus level, the antioxidant supplementation led to a significant increase in the abundance of several taxa, including *norank_f__Muribaculaceae*, *Ruminococcus*, *Sarcina*, *Lachnospiraceae_AC2044_group*, *Eubacterium_ruminantium_group*, *Moryella*, *Anaerovibrio*, *Prevotellaceae_UCG-004*, and *Butyrivibrio* (*p* < 0.05), many of which are associated with fiber degradation and short-chain fatty acid (SCFA) production. Conversely, the abundance of several potential opportunistic pathogens, such as *Empedobacter*, *Paracoccus*, *norank_f__Weeksellaceae*, *Flavobacterium*, *Comamonas*, and *Pseudomonas*, was significantly reduced (*p* < 0.05) (Figure 1d,e). LEfSe analysis also confirmed differentially enriched taxa (Figure 1f,g).

**Figure 2:**
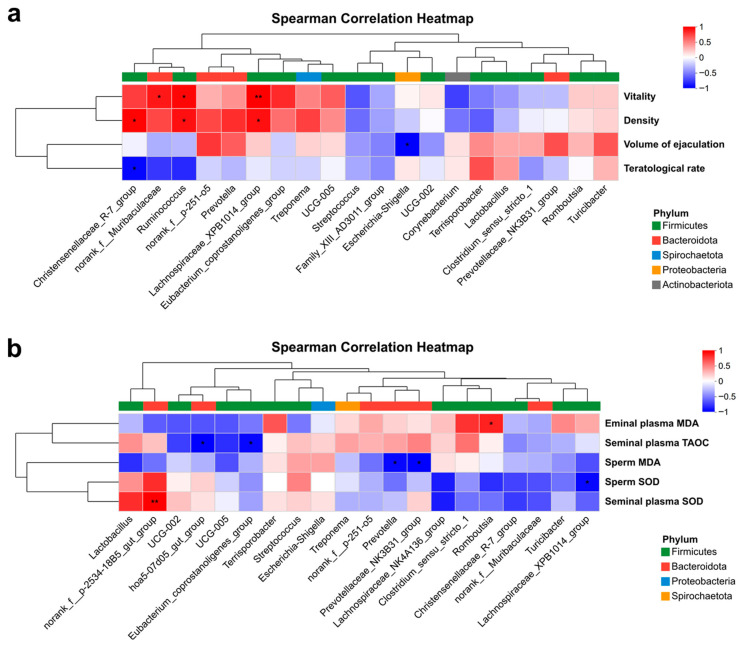
Correlation of fecal microbiota with semen quality and antioxidant parameters in antioxidant-supplemented Ningxiang boars. (**a**). Correlation between fecal microbial communities and semen quality indicators. (**b**). Correlation between gut microbiota and antioxidant indicators. Statistical significance: * *p* < 0.05, ** *p* < 0.01 (Student’s *t*-test).

Correlation analysis revealed several notable associations. Semen vitality showed significant positive correlations with *norank_f__Muribaculaceae* (R = 0.762, *p* = 0.028), *Ruminococcus* (R = 0.810, *p* = 0.015), and *Lachnospiraceae_XPB1014_group* (R = 0.857, *p* = 0.007). Semen density positively correlated with *Christensenellaceae_R-7_group* (R = 0.826, *p* = 0.011), *Ruminococcus* (R = 0.786, *p* = 0.021), and *Lachnospiraceae_XPB1014_group* (R = 0.786, *p* = 0.021). *Christensenellaceae_R-7_group* was negatively correlated with ejaculate volume (R = −0.790, *p* = 0.020), and *Escherichia-Shigella* abundance was negatively associated with teratological sperm rate (R = −0.833, *p* = 0.010) (Figure 2a). Furthermore, *norank_f__p-2534-18B5_gut_group* positively correlated with seminal plasma SOD (R = 0.943, *p* = 0.005), while *Romboutsia* positively correlated with seminal plasma MDA (R = 0.812, *p* = 0.050). *Prevotella* (R = −0.829, *p* = 0.042) and *Prevotellaceae_NK3B31_group* (R = −0.886, *p* = 0.019) were negatively correlated with sperm MDA, and *Lachnospiraceae_XPB1014_group* was negatively correlated with sperm SOD (R = −0.829, *p* = 0.042) (Figure 2b).

**Figure 3:**
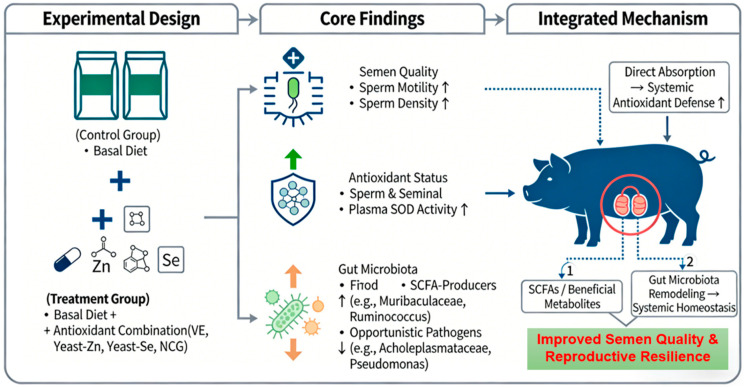
Graphical summary: A dietary antioxidant combination improves semen quality in heat-stressed Ningxiang boars via enhanced antioxidant defense and gut microbiota remodeling.

These results indicate that the dietary antioxidant combination favorably remodels the gut microbiota, a change that may contribute to the improved semen quality observed in Ningxiang boars. The key findings of this study and their proposed underlying mechanisms are summarized in the graphical summary below (Figure 3).

## 4. Discussion

In pig production, semen quality is a critical determinant of breeding efficiency. Dietary antioxidant supplementation has been shown to support normal reproductive function and alleviate oxidative stress [18,19]. This study demonstrates that dietary supplementation with a combination of vitamin E, yeast-derived zinc, yeast-derived selenium and NCG significantly improves semen quality in Ningxiang boars under summer heat stress. The improvements in sperm motility and density were concomitant with enhanced seminal antioxidant defense (elevated SOD activity) and a beneficial remodeling of the gut microbiota.

The antioxidant dosages were selected based on previous research [20,21]. Yeast-derived zinc and selenium exhibit more pronounced biological effects than their inorganic counterparts [22,23,24], NCG, by promoting ammonia metabolism and endogenous arginine synthesis, participates in the arginine–nitric oxide pathway and has been reported to alleviate oxidative damage and improve sperm quality [15,25].

Heat stress is a major cause of seasonal infertility in boars [26]. The increased sperm abnormalities over time in our study indicate that experimental boars experienced significant thermal stress during summer, leading to diminished semen quality. A key mechanism by which heat stress impairs semen quality is through reduced antioxidant activity in seminal plasma, disrupting the balance between ROS production and clearance [27]. Excessive ROS can induce lipid peroxidation of sperm membrane polyunsaturated fatty acids, damage sperm DNA and membrane integrity, and compromise sperm function, ultimately contributing to male infertility [28]. The observed increase in SOD activity, a crucial first-line enzymatic antioxidant, is a key finding [29]. It suggests that the antioxidant combination bolstered the boars’ endogenous defense system against heat stress-induced oxidative damage, providing a plausible mechanism for the improved sperm motility [27,28]. Although no change in MDA was detected, the enhancement of the enzymatic defense likely played a predominant protective role.

Accumulating evidence indicates that nutrition modulates gut microbiota composition, which in turn influences host physiology and health [30,31]. Recent studies highlight the role of gut microbiota in regulating spermatogenesis and semen quality [32,33,34,35]. A novel and significant aspect of our work is the link to the gut microbiome. Antioxidant supplementation altered gut microbial community structure and enriched fiber-degrading and short-chain fatty acid (SCFA)-producing taxa, including *Muribaculaceae*, *Ruminococcus*, *Prevotellaceae_UCG-004*, *Anaerovibrio*, and *Butyrivibrio* [36,37] and exert immunomodulatory and anti-inflammatory effects [38]. Concurrently, we observed a reduction in potential pathogens, including *Acholeplasmataceae*, *Empedobacte*, *Paracoccus*, *Comamonas*, *Pseudomonas*, which may contribute to improved gut homeostasis and indirectly support semen quality [39]. The strong positive correlations between these beneficial microbial taxa and semen quality indicators (vitality, density) suggest that the gut microbiota may be an important mediator in the pathway from diet to improved reproductive performance [32,33,34,35]. SCFAs are known to exert systemic anti-inflammatory and metabolic effects [38], which could create a more favorable physiological state for spermatogenesis.

While this study focused on the combined effect of the antioxidants, future research employing factorial designs could delineate the individual contributions of each component. Furthermore, direct fertility trials (e.g., via artificial insemination) are warranted to confirm the translational impact of the improved semen parameters. The promising effects on the gut-reproductive axis also warrant validation in commercial breeds. Interestingly, the antioxidant and microbial-modulating properties of these components suggest a potential for exploration in semen extenders, a direction for future applied research.

In conclusion, a 60-day dietary supplementation with vitamin E, yeast-derived zinc, yeast-derived selenium, and NCG effectively mitigated heat stress-induced impairment of semen quality in Ningxiang boars. The mechanism appears to involve a dual action: enhancing endogenous antioxidant capacity (SOD activity) and promoting a favorable gut microbiota composition. These findings provide a scientifically grounded, practical strategy for seasonal nutritional management to support reproductive performance in heat-stressed boars. For on-farm application, we recommend initiating this supplementation approximately 60 days prior to the anticipated heat stress period to protect the complete spermatogenic cycle.

## Figures and Tables

**Table 1:** Feed ingredients and nutritional composition (air-dried basis).

Items	Treatment	
	Control (Group B)	Experiment (Group A)
Corn, %	47.00	47.00
Wheat bran, %	10.00	10.00
Flour, %	20.00	20.00
43% Soybean meal, %	9.00	9.00
Fermented soybean meal, %	5.00	5.00
Extruded soybean, %	3.00	3.00
Fish meal, %	3.00	3.00
Soy oil, %	1.00	1.00
Premix ^1^, %	2.00	2.00
Total, %	100.00	100.00
vitamin E, %	-	0.04
yeast-derived zinc, %	-	0.50
yeast-derived selenium, %	-	0.025
N-carbamylglutamate, %	-	0.08
Nutritional level ^2^		
Digestible energy, MJ/kg	14.48	14.48
CP, %	19.60	19.60
Ca, %	0.92	0.92
Total *p*, %	0.65	0.65
Available *p*, %	0.42	0.42
Lys, %	1.39	1.39
Met, %	0.49	0.49
Thr, %	0.87	0.87
Trp, %	0.22	0.22

^1^ The premix supplies the following nutrients per kilogram of diet: 10.00 mg basic copper chloride, 400.00 mg zinc oxide, 330.00 mg ferrous sulfate, 130.00 mg manganese sulfate, 19.00 mg potassium iodide, 6.00 mg sodium selenite, 10,500 IU vitamin A, 3300 IU Vitamin D3, 22.50 IU Vitamin E, 3.00 mg Vitamin K3, 3.00 mg Vitamin B1, 3.00 mg Riboflavin, 15.00 mg d-pantothenic acid, 30 mg Niacin, 0.12 mg biotin, 1.50 mg folic acid, and 500.00 mg choline. ^2^ Nutrient levels are calculated values.

**Table 2:** Semen quality parameters of Ningxiang boars in the control (B) and antioxidant-supplemented (A) groups at baseline (T1), 30 (T2), and 60 (T3) days.

Item	Acquisition Time	Group A	Group B	*p*-Value
volume of ejaculation, mL	T1	254.20 ± 39.83	270.60 ± 26.40	0.740
	T2	267.80 ± 36.30	280.20 ± 35.74	0.814
	T3	303.00 ± 23.86	308.80 ± 36.41	0.897
Density, 10^9^/mL	T1	1.85 ± 0.68	1.23 ± 0.12	0.396
	T2	2.66 ± 0.54	1.62 ± 0.39	0.156
	T3	2.43 ± 0.42	1.17 ± 0.22	0.029
Vitality	T1	0.60 ± 0.11	0.55 ± 0.14	0.789
	T2	0.81 ± 0.05	0.50 ± 0.13	0.053
	T3	0.86 ± 0.04	0.53 ± 0.10	0.013
Teratological rate, %	T1	23.10 ± 3.43	31.08 ± 7.09	0.292
	T2	36.62 ± 6.71	43.36 ± 6.32	0.528
	T3	48.92 ± 5.46	52.29 ± 5.80	0.708

**Table 3:** Antioxidant parameters in sperm and seminal plasma of Ningxiang boars from the third semen collection (Day 60).

Item	Group A	Group B	*p*-Value
seminal plasma MDA	0.43 ± 0.16	0.89 ± 0.55	0.844
sperm MDA	2.92 ± 0.58	2.79 ± 0.30	0.461
sperm SOD	52.08 ± 3.33	17.38 ± 6.62	0.009
seminal plasma SOD	51.05 ± 1.09	47.91 ± 1.43	0.039
seminal plasma TAOC	0.47 ± 0.08	0.50 ± 0.16	0.736

## Data Availability

The original contributions presented in this study are included in the article. Further inquiries can be directed to the corresponding authors.

## References

[1] Sui H., Wang S., Liu G., Meng F., Cao Z., Zhang Y. (2022). Effects of Heat Stress on Motion Characteristics and Metabolomic Profiles of Boar Spermatozoa. Genes.

[2] Schafer F.Q., Buettner G.R. (2001). Redox environment of the cell as viewed through the redox state of the glutathione disulfide/glutathione couple. Free Radic. Biol. Med..

[3] Shiota Yokomizo A., Tada Y., Inokuchi J., Kashiwagi E., Masubuchi D., Eto M., Uchiumi T., Naito S. (2010). Castration resistance of prostate cancer cells caused by castration-induced oxidative stress through Twist1 and androgen receptor overexpression. Oncogene.

[4] Surai P.F., Fisinin V.I. (2015). Selenium in pig nutrition and reproduction: Boars and semen quality—A review. Asian-Australas. J. Anim. Sci..

[5] Surai P.F., Blesbois E., Grasseau I., Chalah T., Brillard J.P., Wishart G.J., Cerolini S., Sparks N.H. (1998). Fatty acid composition, glutathione peroxidase and superoxide dismutase activity and total antioxidant activity of avian semen. Comp. Biochem. Physiol. Part B Biochem. Mol. Biol..

[6] Kohen R., Nyska A. (2002). Oxidation of biological systems: Oxidative stress phenomena, antioxidants, redox reactions, and methods for their quantification. Toxicol. Pathol..

[7] Mruk D.D., Silvestrini B., Mo M.Y., Cheng C.Y. (2002). Antioxidant superoxide dismutase—A review: Its function, regulation in the testis, and role in male fertility. Contraception.

[8] Strzezek J. (2002). Secretory activity of boar seminal vesicle glands. Reprod. Biol..

[9] Zelko I.N., Mariani T.J., Folz R.J. (2002). Superoxide dismutase multigene family: A comparison of the CuZn-SOD (SOD1), Mn-SOD (SOD2), and EC-SOD (SOD3) gene structures, evolution, and expression. Free Radic. Biol. Med..

[10] Brigelius-Flohé R., Maiorino M. (2013). Glutathione peroxidases. Biochim. Et Biophys. Acta.

[11] Agarwal A., Nallella K.P., Allamaneni S.S., Said T.M. (2004). Role of antioxidants in treatment of male infertility: An overview of the literature. Reprod. Biomed. Online.

[12] Li Z., Li Y., Zhou X., Cao Y., Li C. (2017). Preventive effects of supplemental dietary zinc on heat-induced damage in the epididymis of boars. J. Therm. Biol..

[13] Liu Q., Zhou Y., Duan R., Wei H., Jiang S., Peng J. (2017). Lower dietary n-6: n-3 ratio and high-dose vitamin E supplementation improve sperm morphology and oxidative stress in boars. Reprod. Fertil. Dev..

[14] Zhang H., Sun H., Peng A., Guo S., Wang M., Loor J.J., Wang H. (2019). N-carbamylglutamate and l-arginine promote intestinal function in suckling lambs with intrauterine growth restriction by regulating antioxidant capacity via a nitric oxide-dependent pathway. Food Funct..

[15] Ma W., Dang X., Zhang J., Wang C., Li D. (2022). Effects of dietary supplementation of N-carbamylglutamate on the haematology parameters, secondary sexual characteristics and testicular gene expression in roosters. J. Anim. Physiol. Anim. Nutr..

[16] Alswat A.S. (2024). The Influence of the Gut Microbiota on Host Health: A Focus on the Gut-Lung Axis and Therapeutic Approaches. Life.

[17] (2021). Boar liquid semen.

[18] Pintus E., Kadlec M., Jovičić M., Sedmíková M., Ros-Santaella J.L. (2018). Aminoguanidine Protects Boar Spermatozoa against the Deleterious Effects of Oxidative Stress. Pharmaceutics.

[19] Galić I., Dragin S., Stančić I., Maletić M., Apić J., Kladar N., Spasojević J., Grba J., Kovačević Z. (2022). Effect of an Antioxidant Supplement Combination on Boar Sperm. Animals.

[20] Liu Q., Zhou Y.F., Duan R.J., Wei H.K., Jiang S.W., Peng J. (2015). Effects of dietary n-6: n-3 fatty acid ratio and vitamin E on semen quality, fatty acid composition and antioxidant status in boars. Anim. Reprod. Sci..

[21] Wang C., Shang L., Guo Q., Duan Y., Han M., Li F., Yin Y., Qiao S. (2022). Effectiveness and safety evaluation of graded levels of N-carbamylglutamate in growing-finishing pigs. Anim. Nutr. (Zhongguo Xu Mu Shou Yi Xue Hui).

[22] Mahan D.C., Cline T.R., Richert B. (1999). Effects of dietary levels of selenium-enriched yeast and sodium selenite as selenium sources fed to growing-finishing pigs on performance, tissue selenium, serum glutathione peroxidase activity, carcass characteristics, and loin quality. J. Anim. Sci..

[23] López A., Rijsselaere T., Van Soom A., Leroy J.L., De Clercq J.B., Bols P.E., Maes D. (2010). Effect of organic selenium in the diet on sperm quality of boars. Reprod. Domest. Anim. Zuchthyg..

[24] Nitrayova S., Windisch W., von Heimendahl E., Müller A., Bartelt J. (2012). Bioavailability of zinc from different sources in pigs. J. Anim. Sci..

[25] Chen J.Q., Li Y.S., Li Z.J., Lu H.X., Zhu P.Q., Li C.M. (2018). Dietary l-arginine supplementation improves semen quality and libido of boars under high ambient temperature. Anim. Int. J. Anim. Biosci..

[26] Parrish J.J., Willenburg K.L., Gibbs K.M., Yagoda K.B., Krautkramer M.M., Loether T.M., Melo F. (2017). Scrotal insulation and sperm production in the boar. Mol. Reprod. Dev..

[27] Aitken R.J., De Iuliis G.N. (2007). Origins and consequences of DNA damage in male germ cells. Reprod. Biomed. Online.

[28] Agarwal A., Saleh R.A., Bedaiwy M.A. (2003). Role of reactive oxygen species in the pathophysiology of human reproduction. Fertil. Steril..

[29] Yan L., Liu J., Wu S., Zhang S., Ji G., Gu A. (2014). Seminal superoxide dismutase activity and its relationship with semen quality and SOD gene polymorphism. J. Assist. Reprod. Genet..

[30] Wang L., Zou L., Li J., Yang H., Yin Y. (2021). Effect of dietary folate level on organ weight, digesta pH, short-chain fatty acid concentration, and intestinal microbiota of weaned piglets. J. Anim. Sci..

[31] Yang C., Wang M., Tang X., Yang H., Li F., Wang Y., Li J., Yin Y. (2021). Effect of Dietary Amylose/Amylopectin Ratio on Intestinal Health and Cecal Microbes’ Profiles of Weaned Pigs Undergoing Feed Transition or Challenged With Escherichia coli Lipopolysaccharide. Front. Microbiol..

[32] Ding N., Zhang X., Zhang X.D., Jing J., Liu S.S., Mu Y.P., Peng L.L., Yan Y.J., Xiao G.M., Bi X.Y. (2020). Impairment of spermatogenesis and sperm motility by the high-fat diet-induced dysbiosis of gut microbes. Gut.

[33] Zhang C., Xiong B., Chen L., Ge W., Yin S., Feng Y., Sun Z., Sun Q., Zhao Y., Shen W. (2021). Rescue of male fertility following faecal microbiota transplantation from alginate oligosaccharide-dosed mice. Gut.

[34] Zhang P., Feng Y., Li L., Ge W., Yu S., Hao Y., Shen W., Han X., Ma D., Yin S. (2021). Improvement in sperm quality and spermatogenesis following faecal microbiota transplantation from alginate oligosaccharide dosed mice. Gut.

[35] Zhang T., Sun P., Geng Q., Fan H., Gong Y., Hu Y., Shan L., Sun Y., Shen W., Zhou Y. (2022). Disrupted spermatogenesis in a metabolic syndrome model: The role of vitamin A metabolism in the gut-testis axis. Gut.

[36] Olia Bagheri F., Alizadeh A., Gilani M.A.S., Shahhoseini M. (2021). Role of peroxisome proliferator-activated receptor gamma (PPARγ) in the regulation of fatty acid metabolism related gene expressions in testis of men with impaired spermatogenesis. Reprod. Biol..

[37] Lin Y., Wang K., Che L., Fang Z., Xu S., Feng B., Zhuo Y., Li J., Wu C., Zhang J. (2022). The Improvement of Semen Quality by Dietary Fiber Intake Is Positively Related With Gut Microbiota and SCFA in a Boar Model. Front. Microbiol..

[38] Flint H.J., Scott K.P., Louis P., Duncan S.H. (2012). The role of the gut microbiota in nutrition and health. Nature reviews. Gastroenterol. Hepatol..

[39] Sánchez-Alcoholado L., Ordóñez R., Otero A., Plaza-Andrade I., Laborda-Illanes A., Medina J.A., Ramos-Molina B., Gómez-Millán J., Queipo-Ortuño M.I. (2020). Gut Microbiota-Mediated Inflammation and Gut Permeability in Patients with Obesity and Colorectal Cancer. Int. J. Mol. Sci..

